# Building Bridges toward Invasion: Tumor Promoter Treatment Induces a Novel Protein Kinase C-Dependent Phenotype in MCF10A Mammary Cell Acini

**DOI:** 10.1371/journal.pone.0090722

**Published:** 2014-03-05

**Authors:** Kristine S. Klos, Janel K. Warmka, Disa M. Drachenberg, Liang Chang, G. W. Gant Luxton, Cheuk T. Leung, Kathryn L. Schwertfeger, Elizabeth V. Wattenberg

**Affiliations:** 1 Division of Environmental Health Sciences, School of Public Health, University of Minnesota, Minneapolis, Minnesota, United States of America; 2 Department of Genetics, Cell Biology, and Development, University of Minnesota, Minneapolis, Minnesota, United States of America; 3 Department of Pharmacology, University of Minnesota, Minneapolis, Minnesota, United States of America; 4 Department of Laboratory Medicine and Pathology and Masonic Cancer Center, University of Minnesota, Minneapolis, Minnesota, United States of America; University of Birmingham, United Kingdom

## Abstract

The potent tumor promoter 12-*O*-tetradecanoylphorbol-13-acetate (TPA) alters many cellular processes through activation of its receptor protein kinase C (PKC), including gene expression, cell cycle, and the regulation of cell morphology, raising an important question for developing targeted methods to prevent cancer: which effects of TPA are crucial for carcinogenesis? To address this question, we studied TPA action in the 3-dimensional (3D) MCF10A human breast epithelial cell system, which models important features of *in vivo* epithelial tissue including growth constraints, structural organization of cells, and establishment of a basement membrane. MCF10A cells, which are immortalized but nontumorigenic, form hollow, spheroid structures in 3D culture referred to as acini. The development of normal acini requires the tight spatiotemporal regulation of cellular proliferation, polarization, apoptosis, and growth arrest. Treatment of MCF10A acini with TPA caused the appearance of multi-acinar structures. Surprisingly, this phenotype did not involve an increase in cell number or major changes in cell death, and polarization. Instead, live cell and confocal microscopy revealed that TPA stimulates MCF10A acini to aggregate. TPA induces the PKC-dependent production of actin-based protrusions, which leads to the formation of cellular bridges between acini, the clustering of acini, and allows cells to move into adjacent acini. During this process, the integrity of the laminin V basement membrane is disrupted, while E-cadherin-based cell-cell contacts remain intact. Altogether, our results show that under the biochemical and structural constraints of epithelial tissue, as modeled by the 3D MCF10A system, TPA induces a novel PKC-dependent phenotype that resembles local invasion. Of the many effects caused by TPA, these studies highlight the aggressive production of actin-based cellular protrusions as a potentially important event along the pathway to carcinogenesis.

## Introduction

The multi-stage nature of carcinogenesis implies that cells must overcome various types of intra- and inter-cellular barriers to progress toward cancer [1], [2]. Identifying how cells overcome these controls is critical for understanding, and ultimately preventing, the process of carcinogenesis. The prototypical tumor promoter 12-*O*-tetradecanoylphorbol-13-acetate (TPA, also known as phorbol-12-myristate-13-acetate or PMA) is an excellent tool for revealing such mechanisms [3], [4], [5]. TPA was identified as a potent tumor promoter in the multi-stage mouse skin model of carcinogenesis. The first step in this model, called initiation, involves activation of the oncogene Ras by a genotoxic carcinogen [4], [5]. Importantly, activation of Ras alone is not sufficient for tumors to develop, suggesting that biochemical and structural constraints keep the action of this powerful oncogene in check. Tumor development requires subsequent treatment with a tumor promoter, such as TPA [4], [5]. TPA is neither genotoxic nor carcinogenic alone. TPA is a potent direct activator of protein kinase C (PKC), however, suggesting that abnormal activation of PKC may help cells overcome barriers that prevent tumorigenesis [5]. Accordingly, altered expression of PKC is associated with many different types of cancer [5]. Furthermore, activation of the ERBB family of receptor tyrosine kinases, which are frequently over-stimulated in different types of cancer, such as breast cancer, results in the release of diacylglycerol, which is the endogenous activator of PKC [5], [6], [7]. Altogether, this has led to the use of TPA as tool to probe the roles of abnormal activation of protein kinase C in a variety of cell culture systems that are used as models to provide clues to molecular and cellular events that may be important in carcinogensis [8], [9].

PKC is a family of isoenzymes that are key components of signaling pathways that regulate a wide variety of functions, including gene expression, cell cycle, cell death, differentiation, motility, and invasion [5]. Phorbol esters, such as TPA, activate the classical PKC isoenzymes (PKCα, PKCβ, PKCγ) and the novel PKC isoenzymes (PKCδ, PKCε, PKCθ, PKCη). TPA does not activate the atypical PKC isoenzymes (PKCζ and PKCι). PKC isoenzymes can differ in their function and in their tissue-specific expression [10]. This helps explain how TPA can stimulate such a wide range of biochemical and cellular effects in cell culture and *in vivo* models, and why the effects of TPA can differ dramatically depending on the context [8], [11].

The complex action of TPA raises the question of which effect or effects are critical for helping cells advance along the pathway of carcinogenesis. Identifying the important events that occur during early stages of carcinogenesis can aid the development of targeted strategies for preventing cancer. To address this question, we investigated the action of TPA in a three-dimensional (3D) cell culture system that uses human cells to model the cellular organization, signaling, and growth constraints of epithelial tissues [12]. Investigating the action of TPA in a 3D human cell culture model could reveal information about the roles of PKC in carcinogenesis that may have been missed by studies conducted in traditional monolayer tissue culture models and *in vivo* rodent models. We chose the 3D MCF10A human breast epithelial cell system because it recreates important features of *in vivo* epithelial tissue that affect cell signaling, including the spatial organization of cells, cell polarization, and establishment of a basement membrane [12]. MCF10A cells are immortalized, but nontumorigenic [13]. When grown within 3D culture conditions, MCF10A cells form hollow, spheroid structures referred to as acini. The correct formation of acini requires the tight spatiotemporal regulation of cell proliferation, cell polarization, apoptosis, and growth arrest [12]. The 3D MCF10A model has provided insight into how the expression of different oncogenes disrupts the coordination of these basic cellular functions resulting in changes in the morphology of 3D MCF10A structures that correspond to different stages of carcinogenesis [12]. Altogether, these studies suggested that the 3D MCF10A model could provide an integrated picture of the complex action of TPA, and indicate which effects are the most relevant for carcinogenesis.

Our results indicate that TPA stimulates a novel morphological phenotype in the 3D MCF10A model that may provide insight into the role of PKC in carcinogenesis. Surprisingly, within the structural and growth constraints of this model of epithelial tissue, the predominant phenotype does not appear to be due to increases in cell number, or major changes in cell death, and polarization. Rather TPA stimulates the PKC-dependent formation of actin-containing protrusions that lead to the aggregation of individual acini into multi-acinar structures, and allows cells to move into neighboring acini. Altogether, our results highlight the amplified production of actin-based cellular protrusions as a potentially important effect of abnormal activation of PKC during early stages of carcinogenesis.

## Materials and Methods

### Chemicals and Reagents

DMEM/F12 and horse serum were purchased from Life Technologies (Grand Island, NY). Epidermal growth factor, hydrocortisone, cholera toxin, TPA, paraformaldehyde, goat serum, bovine serum albumin, triton-X, ethidium bromide and Tween-20 were purchased from Sigma-Aldrich (St. Louis, MO). Insulin was purchased from Akron Biotech (Boca Raton, FL). Matrigel™ was purchased from BD Biosciences (San Jose, CA). Bisindolylmaleimide 1 was purchased from EMD Chemicals, Inc. (San Diego, CA). Glycine was purchased from Thermo Fisher Scientific, Inc. (Waltham, MA).

### Cell Culture

The MCF10A human breast epithelial cells were the gift of Dr. Ben Ho Park, Johns Hopkins University School of Medicine. The MCF10A cell line [14] is commercially available from ATCC (Manassas, VA). MCF10A cells were maintained in DMEM/F12 supplemented with 5% heat-inactivated horse serum, 20 ng/ml epidermal growth factor, 0.5 µg/ml hydrocortisone, 100 ng/ml cholera toxin, and 10 µg/ml insulin. Cells were plated in 3D culture essentially as described in [13]. Briefly, MCF10A cells were plated onto growth factor reduced Matrigel™ in 8-well chamber slides (5000 cells per well). The day the cells were plated was designated “day 0.” The cultures were fed on days 4, 8, 12, and 16 after plating. For the mixed cell experiment, used to determine if cells can move into adjacent acini, MCF10A cells engineered to express either H2B-mCherry or H2B-GFP were combined and plated in a 1∶1 ratio. The GFP in fixed cultures was detected with a rabbit anti-GFP antibody purchased from Novus Biologicals (Littleton, CO).

### Immunofluorescence

Acini were fixed with 2% paraformaldehyde to detect phospho-Histone H3 and actin, 4% paraformaldehyde to detect GM130, H2B-GFP, H2B-mCherry, and laminin, and methanol/acetone to detect E-cadherin. Anti-phospho-Histone H3 (Ser10) rabbit monoclonal antibody, goat anti-rabbit IgG Alexa Fluor 488 conjugate, and goat anti-mouse Alexa Fluor 488 conjugate were purchased from Cell Signaling Technology (Danvers, MA). Mouse anti-GM130 antibody and mouse anti-E-cadherin were purchased from BD Biosciences (San Jose, CA). Mouse anti-laminin-5 antibody Alexa Fluor 488 conjugate was purchased from EMD Millipore Corporation (Billerica, MA). Alexa Fluor 568 phalloidin, Alexa Fluor 546 goat anti-mouse antibody, and ProLong Gold anti-fade reagent with DAPI were purchased from Life Technologies (Grand Island, NY).

### Confocal Microscopy

Images were captured with either an Olympus FluoView 1000 IX2 Inverted Confocal Microscope or an Olympus FluoView 500 Confocal Laser Scanning Microscope under a 10X (0.4 NA) or 40X (1.3 NA) objective (Olympus; Tokyo, Japan) along with FluoView software (Olympus).

### Ethidium Bromide Uptake

Cultures of acini were incubated in a tissue culture incubator (37°C, 5% CO_2_) for 15 minutes with phosphate buffered saline that contained 1 µM ethidium bromide (EtBr), The EtBr was removed and replaced with phosphate buffered saline. Images were captured with a 10X objective (4 views per well of an 8-well chamber slide) under a rhodamine filter within 1 hour of EtBr exposure. Acini that had at least two cells positive for EtBr were scored positive for cell death.

### Live cell microscopy

On day 4, MCF10A cells were fed in the presence of either vehicle (DMSO) or 10 nM TPA, transferred to a climate controlled chamber (37°C, 5% CO_2_), and imaged every 10 minutes for 24 hours with a Zeiss Axio Observer Z1 (Zeiss, Jena, Germany) and Zen 2011 software (Zeiss) under a 10X (0.5 NA) phase objective. Sixteen total fields were captured, each containing three z-stacks, 25 microns apart. Images were analyzed with FIJI software (ImageJ, National Institutes of Health, USA).

### Immunoblot Analysis

MCF10A cells were plated in monolayer. The following day, vehicle (DMSO) or TPA was added to the media. Cell lysates were prepared after 24 hours later with the following buffer: 50 mM Tris-HCl pH 7.4, 150 mM NaCl, 1% Triton X-100, 0.25% sodium deoxycholate, 1 mM EDTA, 1 mM PMSF, 1 µg/ml aprotinin, 1 µg/ml leupeptin, 20 mM β-glycerophosphate, 1 mM Na_3_VO_4_, 1 mM NaF. Lysates were cleared by centrifugation (14,000×*g*, 10 min, 4°C). Twenty µg of protein was resolved using an 8% SDS-polyacrylamide minigel, and then transferred to Immobilon-P PVDF membrane (Millipore, Bedford, MA). After blocking in TBST/5% milk solution at room temperature for one hour, immunoblots were incubated overnight at 4°C using a rabbit anti-E-cadherin antibody, and then for one hour at room temperature with an HRP-conjugated anti-rabbit antibody. The blots were stripped and reprobed for total ERK1/2 as a loading control. The anti-E-cadherin antibody and the HRP-conjugated anti-rabbit antibody were purchased from Cell Signaling Technology (Danvers, MA). The rabbit anti-ERK1 antibody, which detects both ERK1 and ERK2, was purchased from Santa Cruz Biotechnology, Inc. (Dallas, TX). Immunoblots were visualized using the Pierce SuperSignal West Pico substrate.

### Statistical Analyses

Statistical analyses were performed using GraphPad Prism version 4.0 for Macintosh. Statistical significance was assessed as indicated in the figure legends using a paired t-test, an unpaired t-test, or 2-way ANOVA, and the Bonferroni post-test.

## Results

**Figure 1:**
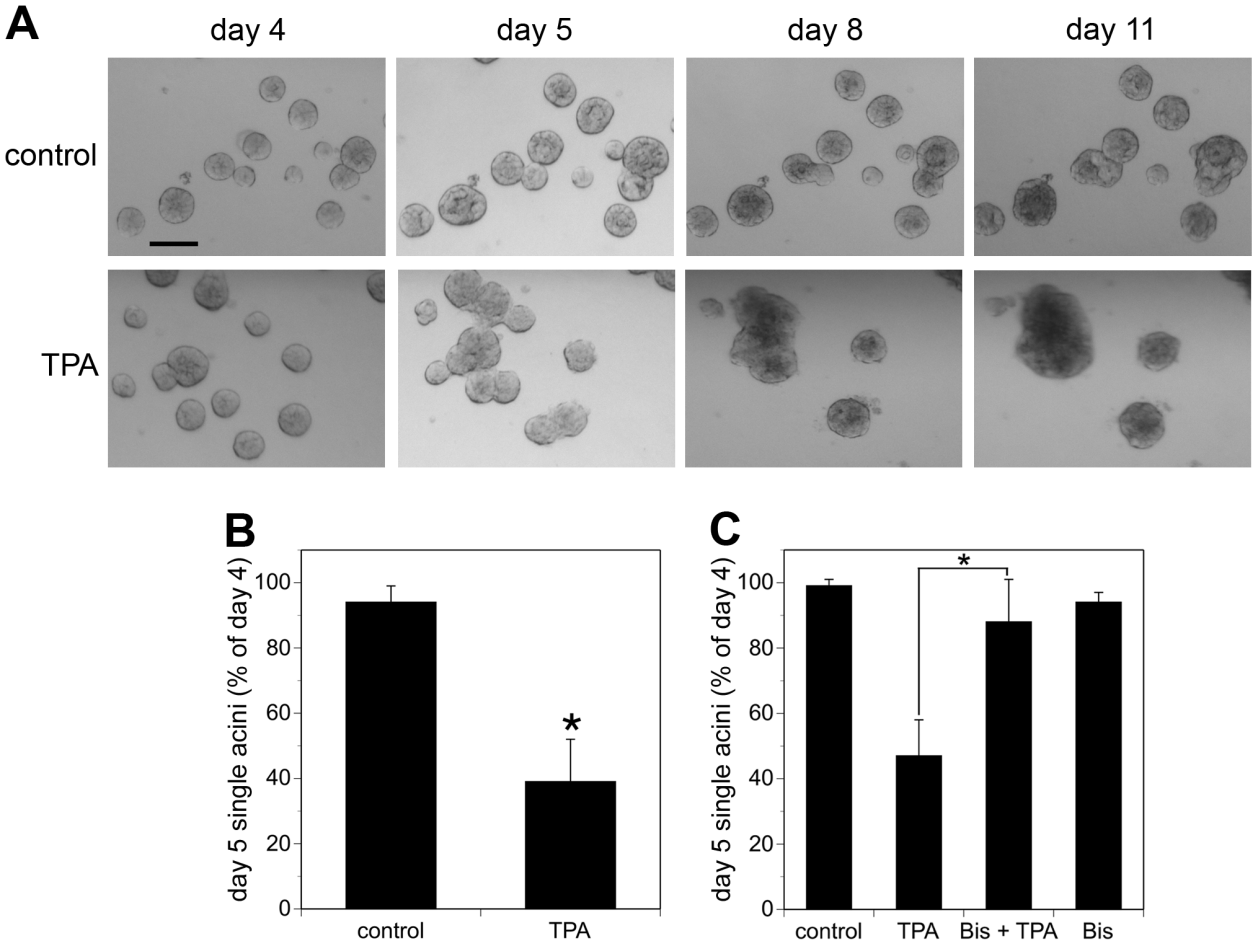
TPA treatment of MCF10A acini results in the appearance of multi-acinar structures. A) Cultures of MCF10A acini were fed on days 4 and 8 in the absence (control, upper panels) or presence of 10 nM TPA (TPA, lower panels). The same fields of acini were analyzed by bright field microscopy (4X) on the indicated days. Bar  = 100 µM. Representative images are shown. B) On day 4, the cultures were fed in the absence (control) or presence of 10 nM TPA (TPA). The same fields of structures were analyzed by light microscopy on day 4 before feeding and again on day 5 approximately 24 hours after the addition of TPA. Fifty structures were analyzed for each condition. The data represent the average of six independent experiments +/− SD. Acini that were touching other acini were scored as multi-acinar. The data are expressed as % single acini on day 5 relative to day 4, calculated as follows: (single acini on day 5/single acini on day 4) X 100. The asterisk denotes a value that was determined to be statistically significantly different from control cultures (p<0.0001) by using a paired t-test. C) On day 4, cultures of MCF10A acini were fed in the absence (control) or presence of 2 µM bisindolylmaleimide I (Bis). TPA or vehicle (DMS0) was added 30 minutes later. The same fields of structures were analyzed by light microscopy on day 4 before feeding and again on day 5 approximately 24 hours after the addition of TPA. At least 50 structures were analyzed for each condition. The data represent the average of three independent experiments +/− SD. The data are expressed as described for B). The asterisk denotes that there is a statistically significant difference between cultures treated with TPA alone and cells treated with both TPA and bisindolylmaleimide (p<0.001) as determined by using a 2-way ANOVA and Bonferroni post-test.

Adding 10 nM TPA to cultures of MCF10A acini four days after plating (referred to as day 4) resulted in the appearance of multi-acinar structures within 24 hours (Fig. 1A, lower panel, compare day 4 and day 5). By day 8, it became difficult to distinguish individual acini within the multi-acinar structures by bright field microscopy (Fig. 1A). Approximately 50% of the structures that appeared as single-acinus on day 4, before addition of TPA, appeared as multi-acinar structures on day 5, 24 hours after the addition of TPA (Fig. 1B). By contrast, in control cultures less than 10% of the structures that were single-acinus on day 4 appeared as multi-acinar structures on day 5 (Fig. 1B). Dose-response studies indicate that 1 nM TPA is not sufficient to induce the appearance of multi-acinar structures (data not shown). In response to 100 nM TPA, approximately 58% the structures appeared as multi-acinar structures within 24 hours, which was close to the range of responses to 10 nM TPA (Fig. 1B and 1C). Therefore, 10 nM TPA was used for the remainder of the studies. Finally, pretreating the cultures with 2 µM bisindolylmaleimide I, a concentration that inhibits TPA-stimulated activation of several isoenzymes of PKC [15], [16], [17], blocked the TPA-induced formation of multi-acinar structures (Fig. 1C).

**Figure 2:**
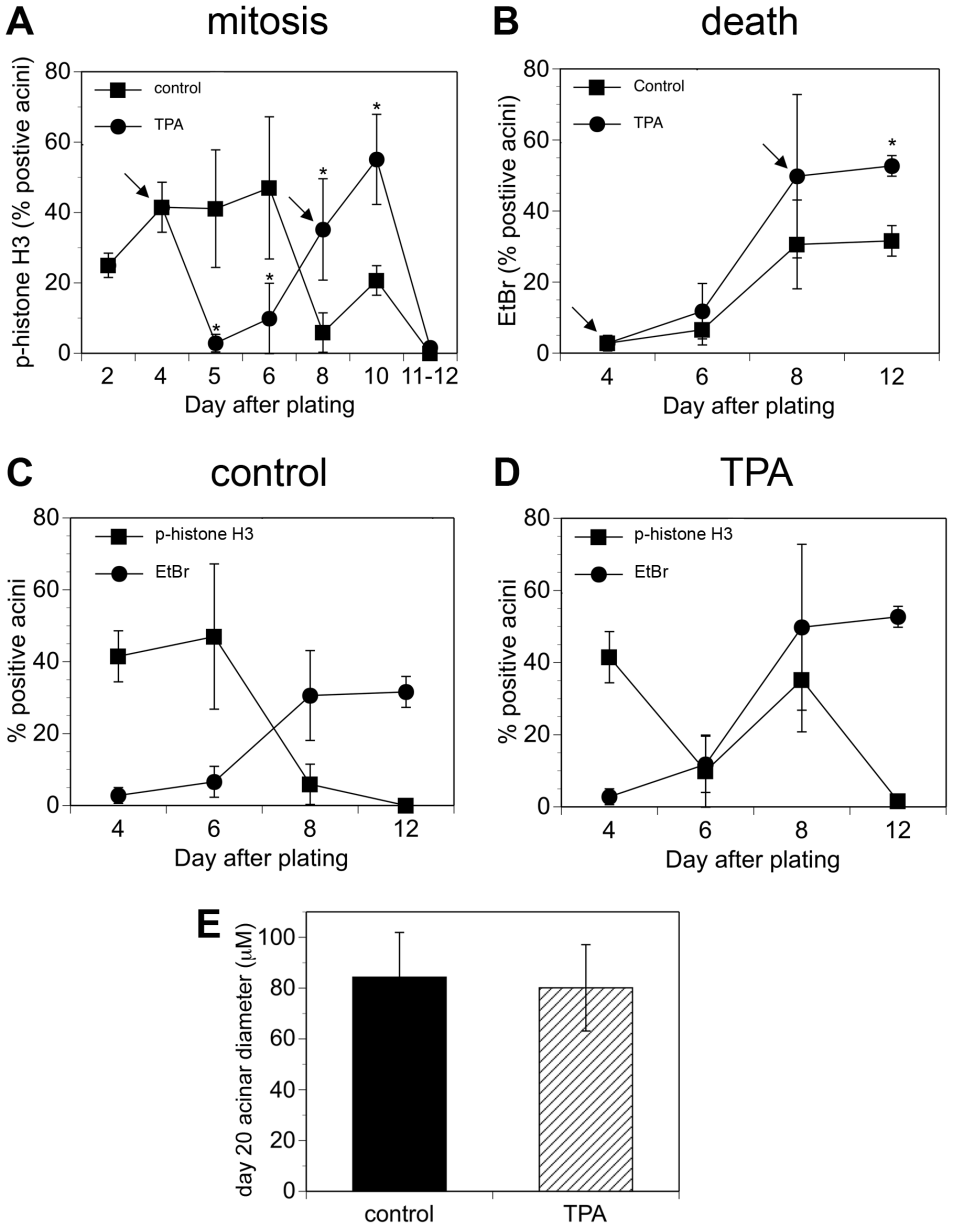
TPA disrupts the temporal coordination between mitosis and cell death. Cultures of MCF10A acini were fed on days 4 and 8 (indicated by the arrows) in the absence (squares) or presence of 10 nM TPA (circles). The scoring on day 4 was conducted prior to feeding or adding TPA. The scoring on day 8 was conducted on cells that were fed in the presence or absence of TPA on day 4. The scoring for the subsequent days was conducted on cells that were fed in the presence or absence of TPA on days 4 and 8. A) On the days shown, the cultures were fixed and then stained to detect phospho-histone H3 as a marker of mitosis. Acini that were positive for at least one cell were scored as positive. On average, at least 100 acini were examined for each data point. Each point on the graph represents the average of at least three independent experiments +/− SD, except Day 11–12, which represents the average of two data points, one from Day 11 (control  = 0, TPA  = 1.2%) and one from Day 12 (control  = 0 and TPA  = 2%). B) On the days shown, ethidium bromide (EtBr) was added to the cultures; uptake of ethidium bromide was used as a marker of cell death. Acini that were positive for at least two cells were scored as positive. At least 190 acini were examined for each data point. The asterisk denotes that there is a statistically significant difference between TPA-treated cultures and control cultures (p<0.05) as determined by using a by using an unpaired t-test. Graphical representation of both mitosis (squares) and death (circles) is shown for C) control acini and D) TPA-treated acini. E) Cultures of MCF10A acini were fed on days 4, 8, 12, and 16 in the absence (control, filled bar) or presence of 10 nM TPA (hatched bar). Diameters at the widest point were measured for single acini on day 20. The average diameters (microns) +/− SD are shown (n = 200).

The appearance of multi-acinar structures could be due to the over-stimulation of proliferation, as occurs with ERBB2 activation in the 3D MCF10A system [12]. Because TPA can stimulate proliferation, depending on the cell type [11], we determined if the multi-acinar phenotype was due to a major increase in cell number. We monitored phospho-histone H3, which is a marker of mitosis, over the course of 12 days (Fig. 2A). Interestingly, TPA had quite different effects on mitosis depending on the stage of acinus formation. We added TPA to cultures of MCF10A acini when they were fed on days 4 and 8 (Fig. 2A, arrows). When we added TPA on day 4, mitosis was blocked within 24 hours and remained low through day 6 (Fig. 2A, circles). By contrast, control cultures maintained a high rate of mitosis through day 6 (Fig. 2A, squares). Mitosis in TPA-treated cultures increased dramatically between days 6 and 10, even after cells were retreated with TPA on day 8 (Fig. 2A, circles). In control cultures, mitosis was minimal by day 8, although it increased slightly by day 10 after refeeding (Fig. 2A, squares). By day 12, mitosis was minimal in both control and TPA-treated cultures (Fig. 2A). The time course of phospho-histone H3 staining in control cultures is consistent with the results of others who showed a high rate of cell proliferation in MCF10A acini through day 4 [18], and a decrease in cell cycle activity between days 6 and 12 [19]. The phospho-histone H3 staining indicates that TPA stimulates a block in mitosis at early highly proliferative stages of acinus formation. Mitosis eventually resumes, and interestingly is not blocked by TPA treatment at later stages of acini development (compare day 10 to day 6). The observation that TPA blocks mitosis at early stages of acinus development is consistent with our observation that acini do not form when MCF10A cells are plated in the presence of TPA (data not shown). It also indicates that the multi-acinar structures that form within 24 hours when TPA is added on day 4 are not caused by a major increase in cell number (see Fig. 1, day 5, lower panel).

Next, we determined whether the large irregular structures that appear by day 11, in cultures treated with TPA on days 4 and 8, were caused by changes in cell death and cell polarization (see Fig. 1, day 11, lower panel). The time course of cell death was similar in both control and TPA-treated cultures, although cell death appeared modestly higher in TPA-treated cultures on day 12 (Fig. 2B, compare circles that represent TPA, to squares that represent control). We monitored cell death by measuring the uptake of ethidium bromide into the cells; ethidium bromide is excluded by live cells, which have intact membranes, but taken up by dead cells, which have lost membrane integrity [19], [20]. Cell death was low in both control and TPA-treated cultures through day 6, it rose dramatically by day 8, and remained elevated through day 12, although by day 12, cell death was higher in TPA-treated cultures than control cultures (Fig. 2B). In control cultures, mitosis and cell death are clearly coordinated (Fig. 2C). During the early phase of acinus formation there is a high rate of mitosis (Fig. 2C, squares) and low rate of cell death (Fig. 2C, circles). During the late phases of acinus formation mitosis decreases (Fig. 2C, squares) and cell death increases (Fig. 2C, circles) as the acini go into growth arrest and hollow out. In TPA-treated cultures, the timing of cell death is similar to that in control cultures (Fig. 2D, circles), even though the timing of mitosis is disrupted, such that it ceases, resumes, and then decreases again (Fig. 2D, squares). This indicates that TPA can disrupt the temporal regulation of mitosis without affecting the temporal regulation of cell death. Despite the differences in the temporal regulation of mitosis, the sizes of single acini in both control and TPA-treated cultures were similar by day 20 (Fig. 2E). These data indicate that under the constraints of 3D culture, TPA does not disrupt growth arrest.

**Figure 3:**
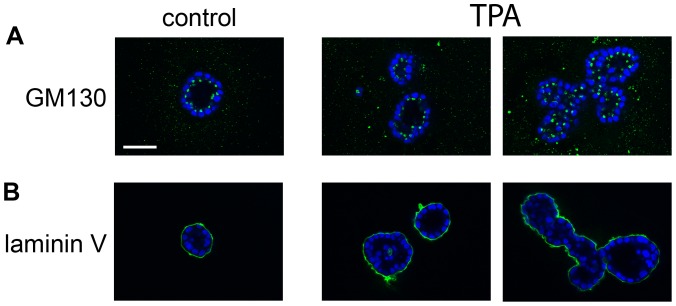
TPA does not alter cell polarization. Cultures of MCF10A acini were fed on days 4, 8, 12, and 16 in the absence (control) or presence of 10 nM TPA (TPA). The cultures were fixed on day 18 and then stained with A) an anti-GM130 antibody (green) and DAPI to detect nuclei (blue), or B) an anti-laminin antibody (green) and DAPI (blue). The images were captured by confocal microscopy (40X). Bar  = 50 microns.

Another important step in acinus formation in the MCF10A model is cell polarization, which is marked by orientation of the Golgi apparatus toward the lumen of the acini, as marked by the GM130 protein (Fig. 3A, green, left panel), and basal deposition of Laminin V (Fig. 3B, green, left panel) [12]. By day 18, MCF10A cells in TPA-treated acini appeared to be polarized (Fig. 3 middle, and right panels). Acini from control cultures are typically hollow by day 18, with GM130 staining oriented toward the lumen (Fig. 3A, green, left panel), and the periphery outlined by laminin V staining (Fig. 3B, green, left panel). Although the multi-acinar structures formed in response to TPA have an irregular shape, GM130 staining still appears oriented toward the lumen (Fig. 3A, green, right panel). Laminin V staining outlines the periphery of the entire multi-acinar structure, which provides supporting evidence that TPA treatment does not disrupt the apico-basal polarization of the cells (Fig. 3B, green, right panel). TPA-treated acini that remain singular also display GM130 staining oriented toward the lumen (Fig. 3A, green, middle panel), and laminin V that outlines the acinus (Fig. 3B, green, middle panel). TPA-treated acini appear to have large areas that are hollow, but some cells are also present within the lumen (Fig. 3, lower middle and right panels). This suggests that in TPA-treated acini, cell death may not totally compensate for cell proliferation. Altogether these data indicate the large, multi-acinar structures that appear in TPA-treated cultures are not caused by an increase in cell number or major changes in the regulation of cell death, polarization, and growth arrest.

**Figure 4:**
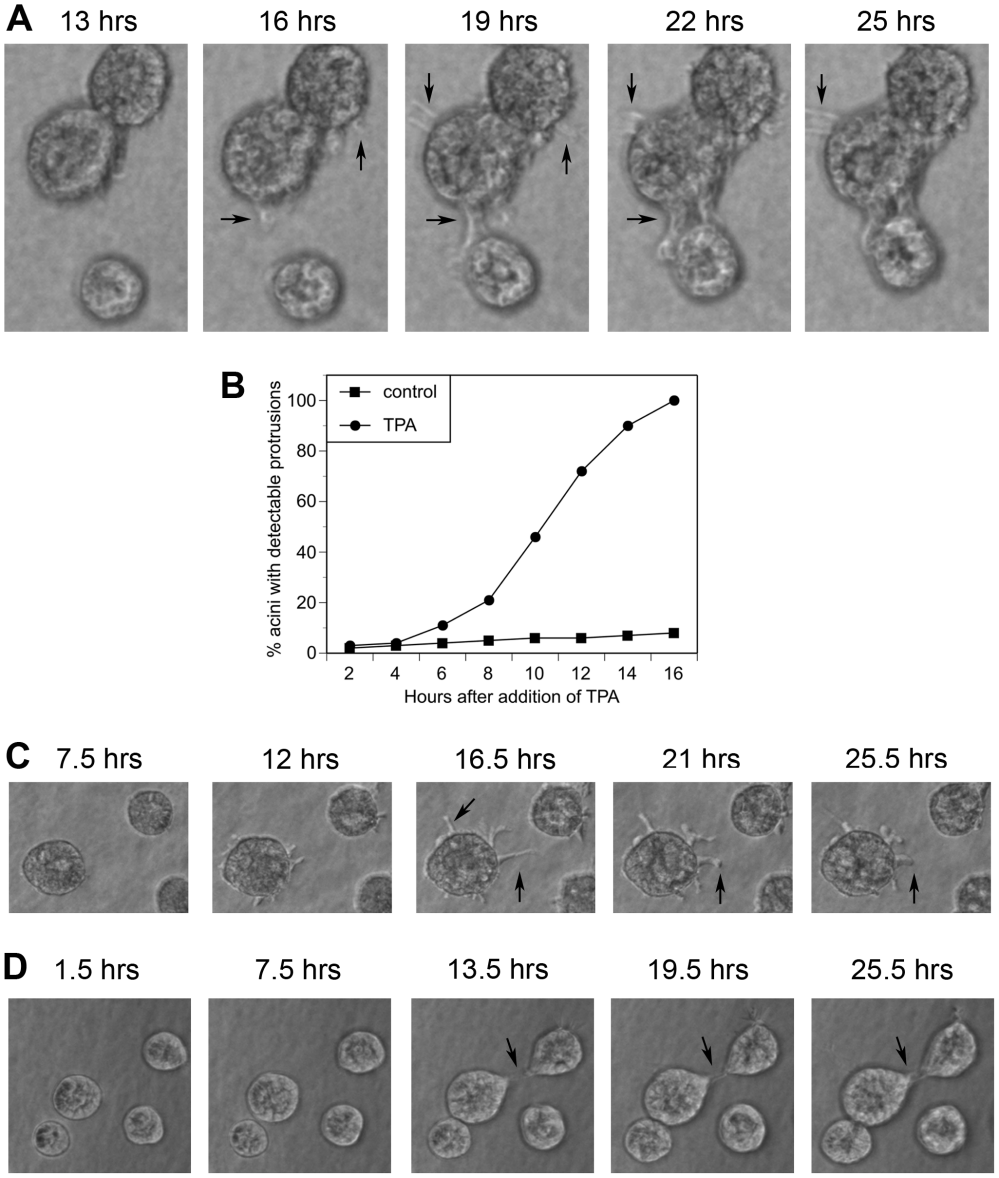
TPA stimulates the formation of protrusions and the aggregation of acini. Cultures of MCF10A acini were fed on day 4 in the absence (control) or presence of 10 nM TPA (TPA). Real time imaging was started 1.5 hours after addition of either vehicle (Video S1, supporting information) or TPA (Video S2, supporting information) and continued for approximately 24 more hours. Images were captured every 10 minutes. A) Images of acini that aggregated captured 13, 16, 19, 22, and 25 hours after addition of TPA. In this field, protrusions appeared by 16 hours, bridges formed by 19 hours, and acini aggregated by 25 hours. B) The real time images from 101 control acini and 104 TPA-treated acini were analyzed to determine the percentage of acini with detectable protrusions over time. C) Images of an acinus that remained single captured at the indicated times after addition of TPA. D) Images of control acini captured at the indicated times after addition of vehicle. Arrows indicate protrusions. Representative images are shown.

**Figure 5:**
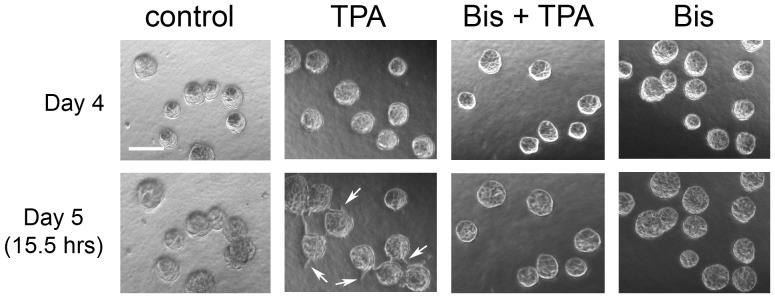
PKC is required for TPA to induce the formation of protrusions. On day 4, cultures of MCF10A acini were fed in the absence (control) or presence of 2 µM bisindolylmaleimide I (Bis). TPA or vehicle was added 30 minutes later. Images were captured by phase contrast microscopy (4X) on day 4 before treatment and again 15.5 hours after addition of TPA. Bar  = 100 µM. Arrows indicate protrusions. Representative images are shown.

Another possible explanation for the appearance of multi-acinar structures in TPA-treated cultures is that TPA stimulates the acini to aggregate. Live cell imaging indicated that TPA does induce individual acini to cluster (Video S1 and Video S2, supporting information and Fig. 4). We observed control cultures (Video S1, supporting information) and TPA-treated cultures (Video S2, supporting information) by live cell imaging starting on day 4, approximately 1.5 hours after adding TPA or vehicle (DMSO), and continued for approximately 24 more hours. TPA stimulated the production of protrusions, which led to the formation of cellular bridges between neighboring acini, which was followed by their aggregation (Fig. 4A). We detected protrusions on 3% of the acini by two hours after addition of TPA (Fig. 4B). Protrusions appeared on 50% of the acini between 10 and 11 hours after addition of TPA, and by 16 hours protrusions appeared on all of the acini (Fig. 4B). Control cells had far fewer detectable protrusions (Fig. 4B). By 16 hours after treatment with vehicle, we detected protrusions on 8% of control acini (Fig. 4B), which increased to only 11% by 21 hours (data not shown). In the TPA-treated cultures, the protrusions extended in all different directions from the acini, not only in the direction of close neighboring acini (Fig. 4C). Acini that did not aggregate still produced protrusions, although the protrusions eventually retracted (4C, compare 16.5 hrs to 25.5 hrs). Finally, acini that were treated with bisindolylmaleimide I prior to treatment with TPA did not produce protrusions, form bridges, or aggregate (Fig. 5).

The multi-acinar structures observed in control cultures appear to result from neighboring acini that come in contact with each other as they increase in size (Video S1, supporting information and Fig 4D, lower left region of panels). In the rare event when we detected protrusions extending toward neighboring control acini, we did not observe aggregation (Fig. 4D). For example, Figure 4A shows protrusions forming 16 hours after TPA treatment; three hours later bridges are forming and aggregation has begun (19 hour time point), as indicated by the shifting up of the lower acini. By contrast, although Figure 4D shows a protrusion forming in a control acinus by 13.5 hours, even 12 hours later the acini have not clustered (Fig. 4D, upper right). This suggests that even if control acini have the ability to form protrusions, and perhaps bridges between acini, TPA treatment greatly accelerates and increases the aggressiveness of this process.

**Figure 6:**
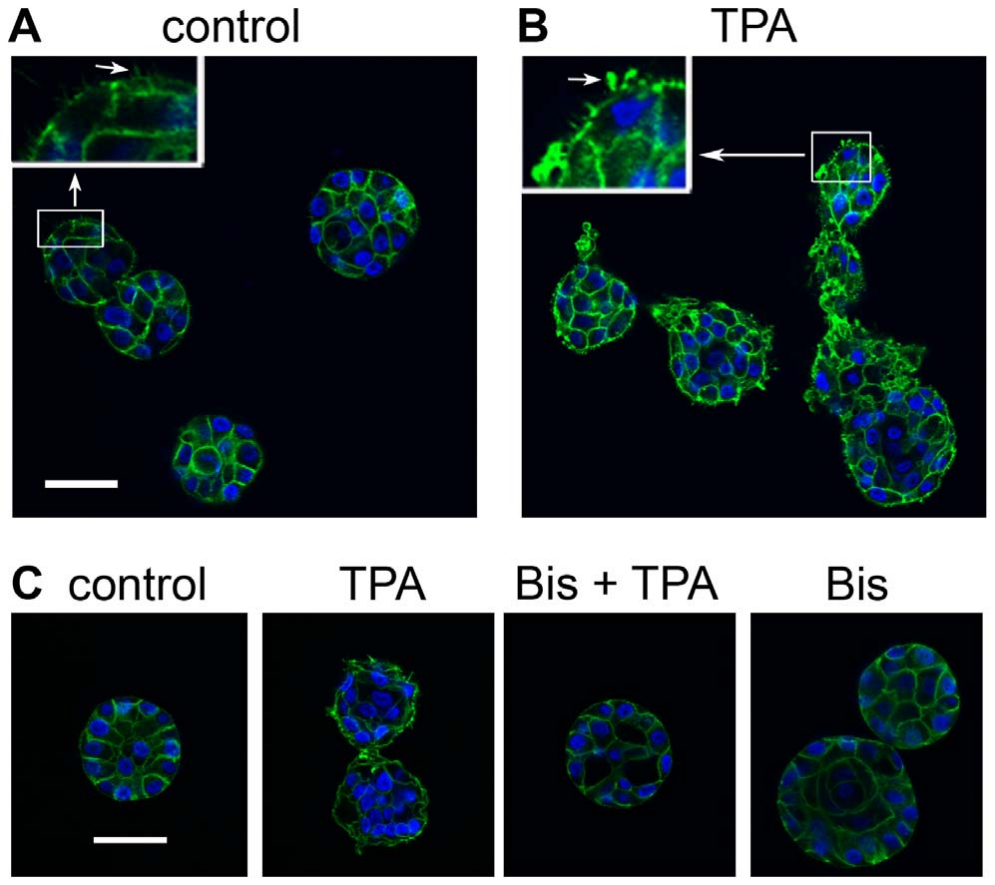
The protrusions formed in response to TPA contain actin. On day 4, cultures of MCF10A acini were fed in A) the absence or B) the presence of 10 nM TPA (TPA). The cultures were fixed 15 hours after the addition of TPA and stained with phalloidin to detect actin (green) and DAPI to detect nuclei (blue). Images were captured by confocal microscopy (40X). Inserts show regions (in boxes) that were digitally enlarged to the same extent to compare protrusions (arrows in inserts) formed in A) control and B) TPA-treated cultures. C) On day 4, cultures of MCF10A acini were fed in the absence (control) or presence of 2 µM bisindolylmaleimide I (Bis). TPA or vehicle (control) was added 30 minutes later. The cultures were fixed 15 hours after the addition of TPA, and then stained to detect actin (green) and nuclei (blue) as described above. Bar  = 50 µM. Representative images are shown.

Various types of cellular protrusions contain actin [21], which led us to examine the actin content of the protrusions formed in response to TPA (Fig. 6). We added TPA to the cultures of acini on day 4, fixed them 15 hours later, and then stained them with phalloidin to detect actin (green) and DAPI to detect nuclei (blue). At 40× magnification, the peripheries of control acini appear smooth (Fig. 6A), although digital enlargement of the images revealed the presence of tiny actin-containing protrusions (Fig. 6A, upper left inset, arrow). By contrast the peripheries of TPA-treated acini appear rough and spiky at 40× magnification (Fig. 6B). The actin-containing protrusions formed in response to TPA were much larger than those of controls, and appear in various shapes and sizes (Fig. 6B). The TPA-induced protrusions contain actin whether the acini aggregate (Fig. 6B, right structure) or remain singular (Fig. 6B, left and middle structures). Pretreatment of the acini with bisindolylmaleimide I blocked the TPA-induced formation of actin-containing protrusions (Fig. 6C), which is consistent with the results obtained with phase contrast imaging of the protrusions (Fig. 5).

**Figure 7:**
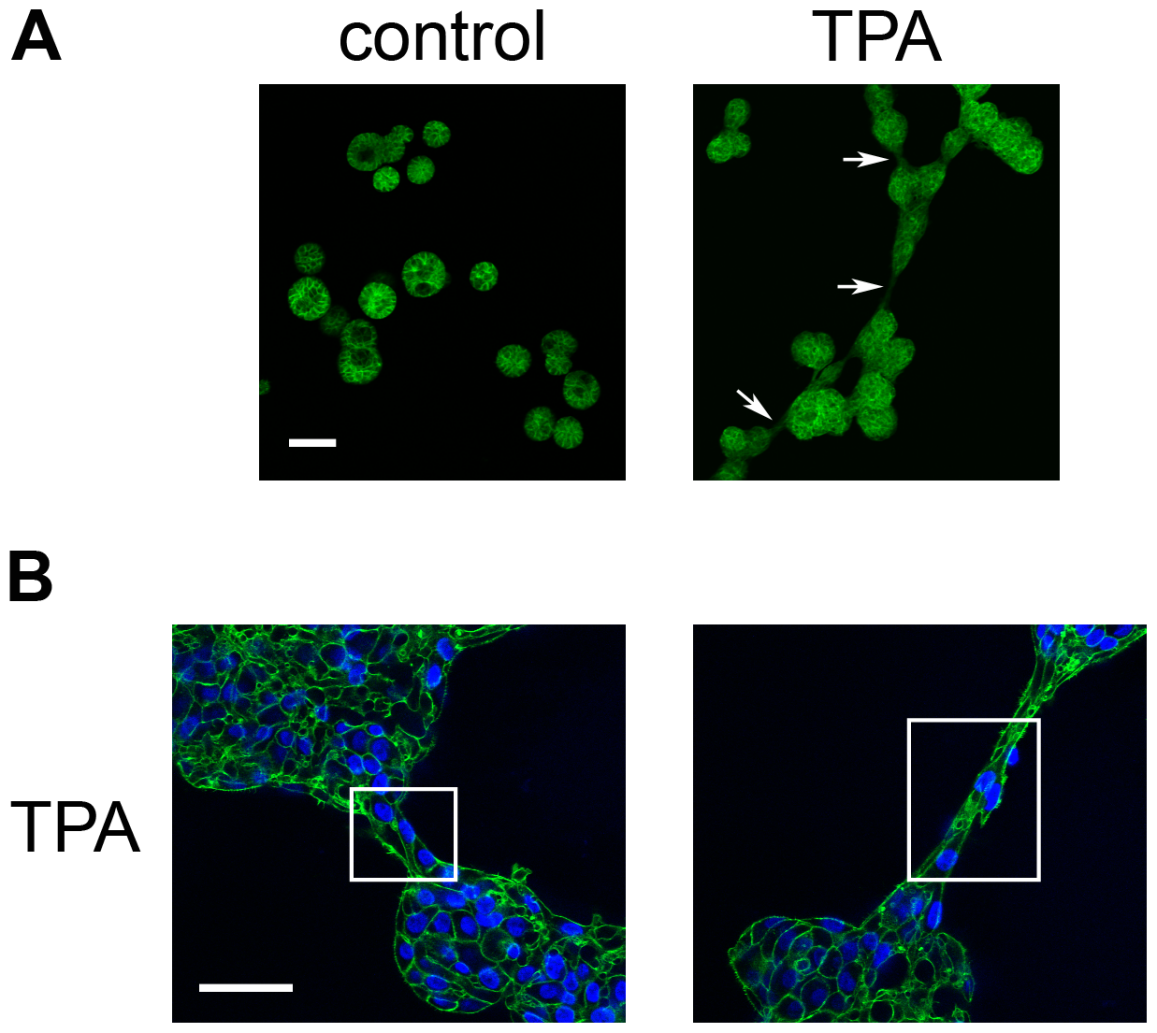
The bridges formed between acini in TPA-treated cultures contain actin. On day 4, cultures of MCF10A acini were fed in the absence (control) or presence of 10 nM TPA (TPA), and fixed 21 hours later. A) Acini stained with phalloidin to detect actin (green) (10X); bar  = 100 µM. Arrows indicate bridges. B) Acini stained with phalloidin and DAPI to detect nuclei (blue) (40X); bar  = 50 µM. Boxes have been drawn around bridges. Representative images are shown.

Live cell imaging and staining with phalloidin indicate that the bridges that form between TPA-treated acini begin as membrane protrusions that contain actin (Fig. 4A and Fig. 7A, arrows). As the bridges develop, however, they can eventually be built out of cells, as indicated by the presence of nuclei stained by DAPI (Fig. 7B, boxes).

**Figure 8:**
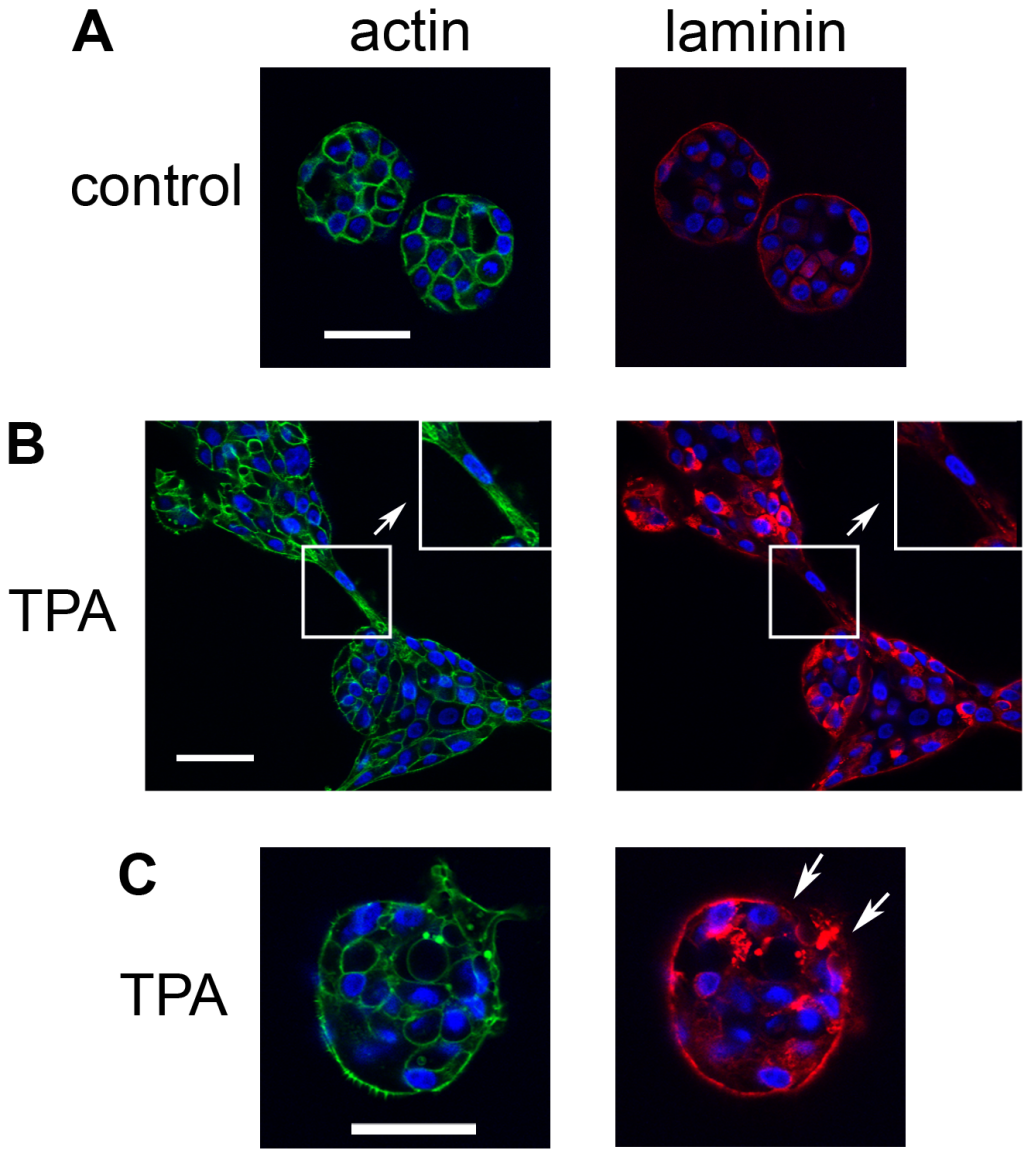
Laminin staining is disrupted at the bridges between acini. On day 4, cultures of MCF10A acini were fed in the A) absence or B) and C) presence of 10 nM TPA. The cultures were fixed 23 hours later and stained with DAPI to detect nuclei (blue) and either phalloidin to detect actin (green, left panels) or an anti-laminin antibody (red, right panels) (40X). Bar  = 50 µM. Inserts in B) show regions that were digitally enlarged to show areas of the bridge (in boxes) that have irregular laminin staining (red, right panel); actin staining for the same region is also shown (green, left panels). Arrows in C) show disrupted laminin staining (red, right panel). Representative images are shown.

**Figure 9:**
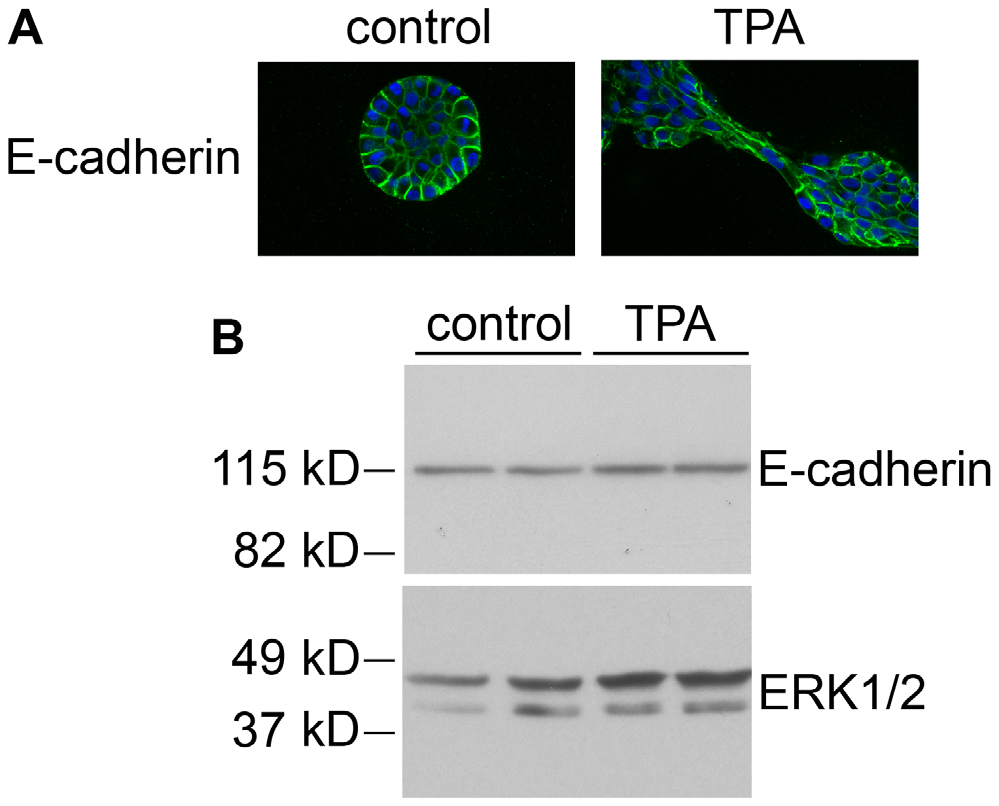
E-cadherin remains intact in TPA-treated acini. A) On day 4, cultures of MCF10A acini were fed in the absence (control) or presence of 10 nM TPA. The cultures were fixed 23 hours later and stained with an anti-E-cadherin antibody (green), and DAPI to detect nuclei (blue) (40X). Bar  = 50 µM. B) MCF10A cells grown in monolayer were treated for 24 hours in the absence (control) or presence of 10 nM TPA (TPA). Whole cell lysates (20 µg) were analyzed by immunoblot for E-cadherin (upper panel), and for ERK1/2 as a loading control (lower panel). Representative images are shown.

To further characterize the bridges, we stained the acini to detect laminin V, which MCF10A cells grown in 3D culture secrete to form a basement membrane (Fig. 8) [12], [13]. In control cultures, laminin V staining appeared as a relatively regular, continuous outline of the periphery of the acini (Fig. 8A, right panel). Laminin V was also detected in the cytoplasm at this early phase in acinus development, although it was not detected in the cytoplasm at later stages (Fig. 3B). In TPA-treated cultures, the integrity of the laminin layer was disrupted at the bridges that formed between acini, such that it did not appear as a distinct outline of the cells in contact with the matrix (Fig. 8B, right panel, box and inset). The laminin layer was also disrupted at the site of large protrusions in TPA-treated acini that remained singular (Fig. 8C, right panel, arrows). Although the laminin layer is disrupted when the acini first aggregate, Figure 3B shows that the integrity of the laminin layer is eventually restored in TPA-treated cultures. Finally, we stained the acini to detect E-cadherin, a transmembrane protein that plays a major role in cell-cell adhesion, and is important for maintaining the structural integrity of the acini [22]. Although the TPA-treated acini generally appear more irregular and disorganized than control acini, the E-cadherin staining appears to remain intact even within the bridges that form between acini (Fig. 9A). Consistent with this observation, TPA did not stimulate a decrease in E-cadherin levels in monolayer cultures of MCF10A cells (Fig. 9B). These data suggest that when the bridges form between acini, the integrity of the basement membrane is lost, but the cells maintain their cell-cell connections.

**Figure 10:**
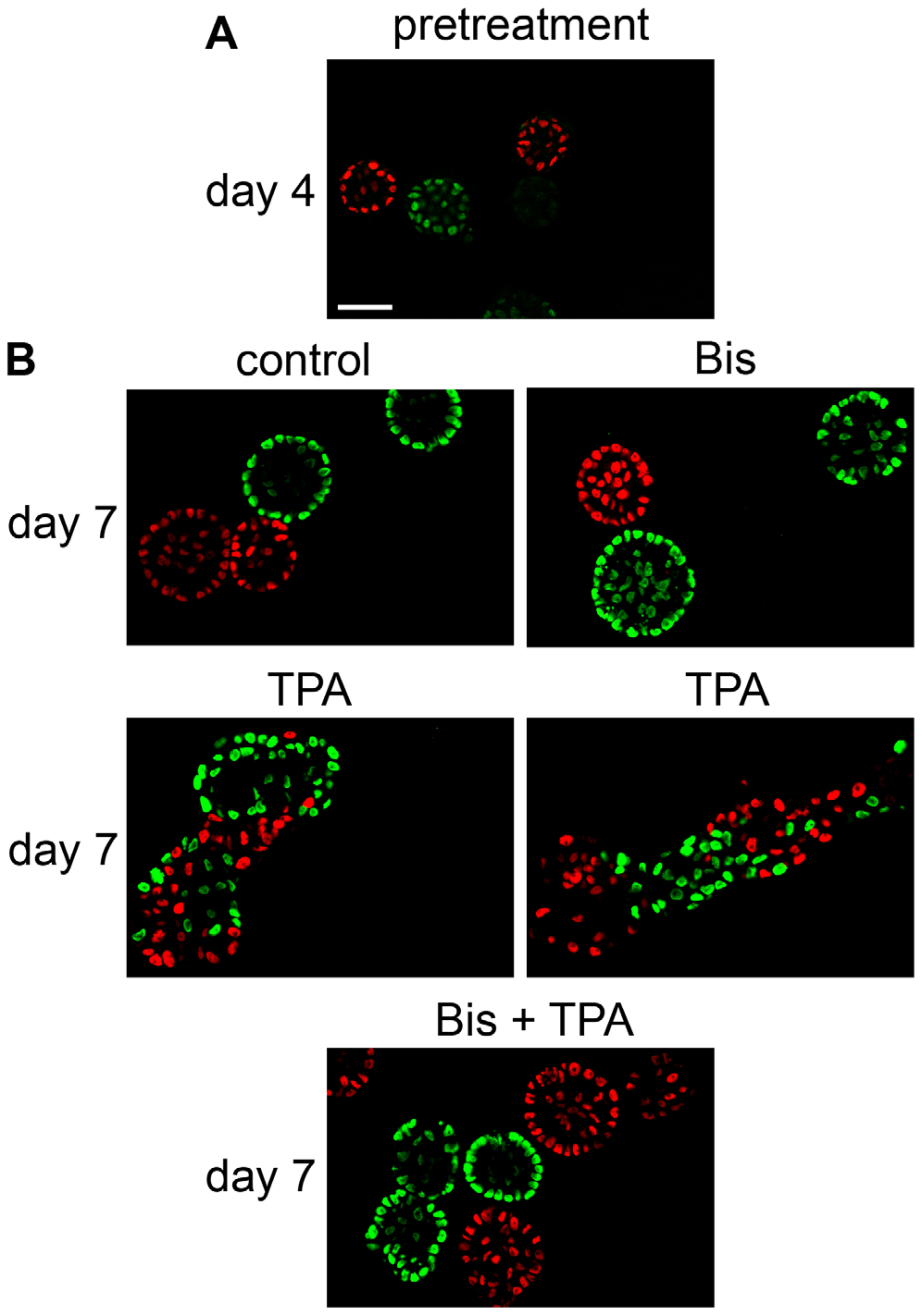
TPA stimulates cells to move into neighboring acini. MCF10A cells engineered to express either H2B-GFP (green) or H2B-mCherry (red) were mixed and plated in a 1:1 ratio. On day 4, cultures of MCF10A acini were fed in the absence (control) or presence of 2 µM bisindolylmaleimide 1 (Bis). TPA or vehicle (control) was added 30 minutes later. A) Images of the cultures of acini on day 4 before feeding (pretreatment). B) Images of the cultures of acini on day 7 (40X). Bar  = 50 µM.

The observation that cells can be present in the bridges between acini suggests that TPA induces a process that may lead to the movement of cells into neighboring acini. To test this, we plated MCF10A cells engineered to express H2B-GFP (green) together with MCF10A cells engineered to express H2B-mCherry (red) (Fig. 10). Each MCF10A acinus develops from a single cell [13]. Accordingly, Figure 10A shows that on Day 4, before treating with TPA, the acini appear green or red. On day 7, control acini and acini treated with bisindolylmaleimide I alone (Bis) still appeared green or red (Fig. 10B, upper panels). Interestingly, by day 7 we found multi-acinar structures in the TPA-treated cultures that were a mixture of both green and red cells (Fig. 10B, middle panels). Acini treated with bisindolylmaleimide I prior to addition of TPA appeared red or green, however (Fig. 10B, lower panels). These results indicate that TPA stimulates the ability of cells to move into neighboring acini through a process that requires PKC.

## Discussion

Under the constraints of epithelial tissue architecture modeled by the 3D MCF10A culture system, the tumor promoter TPA induces a novel PKC-dependent phenotype that resembles steps toward cellular invasion into adjacent tissues. The multitude of effects resulting from TPA-induced activation of PKC, including changes in gene expression, proliferation, death, and changes in cell morphology [11], can obscure which biochemical targets and pathways are key to carcinogenesis. We used the 3D MCF10A system to help indicate which effects of TPA help cells overcome the biochemical and structural controls imposed by epithelial tissue structure, and thus which effects might permit cells to advance along the pathway of carcinogenesis. The most striking change stimulated by TPA in this system is the PKC-dependent production of actin-based protrusions. The protrusions lead to the formation of bridges between different acini, bringing them together to form large, disorganized, multi-acinar structures, and allowing cells to move into adjacent acini. Importantly, we found that although TPA does disrupt the temporal coordination of mitosis and cell death during MCF10A acini formation, the balance between mitosis and death is largely maintained by the cell-cell interactions within the 3D architecture. Altogether, these results highlight the PKC-dependent formation of protrusions as TPA-induced actions that are potentially important for the process of carcinogenesis.

TPA induces a phenotype that differs significantly from the invasive phenotype induced by over-stimulation of colony stimulating factor receptor (CSF-1R) in the 3D MCF10A system [23]. Over-stimulation of CSF-1R in the 3D MCF10A system results in the relocalization of E-cadherin, the disruption of cell-cell adhesions, and release of single cells into the matrix [23]. By contrast, TPA does not cause major changes in E-cadherin localization or protein levels, the cell-cell contacts remain intact, and although the cells move into adjacent acini, we did not observe the release of single cells into the matrix. The TPA-induced phenotype may represent an early phase of local invasion. This would be consistent with abnormal activation of PKC resulting in tumor promotion in other models, which represents an early stage in carcinogenesis [5].

Studies conducted in breast cancer cells suggest that abnormal activation of PKC may also contribute to invasion at later stages of carcinogenesis. For example a recent study suggests that PKC is a key regulator of breast cancer stem cells that have an aggressive phenotype [24]. Accordingly, over-expression of PKCα in MCF-7 breast cancer cells increases their ability to metastasize in mice [25]. Furthermore, expression and activation of ERBB2 in MCF-7 cells and MDA-MB-435 breast cancer cells induces invasion through a pathway that involves PKCα [26], [27]. Intriguingly, expression and activation of ERBB2 in MCF-7 cells also results in the formation of actin-containing protrusions [26]. This suggests that PKC-dependent formation of actin-containing protrusions may be important in both early and late stages of invasion.

PKC can affect the regulation of actin both directly, by phosphorylation of actin regulators, and indirectly, as a mediator in signal transduction [28]. Among the substrates of PKC that are directly involved in regulating actin and play a role in invasion is fascin [28], [29]. PKC can also mediate the regulation of actin by EGF through the phosphorylation of α4β6 integrin [30]. Two likely PKC isoenzymes for mediating the effects of TPA on the regulation of actin in the 3D MCF10A system are PKCδ and PKCε. Our preliminary data indicate that MCF10A cells express the following novel and classical isoenzymes of PKC that can be activated by TPA, PKCα, PKCδ, PKCε, PKCθ, and PKCη. Of these PKC isoenzymes, bisindolylmaleimide I inhibits PKCα, PKCδ, and PKCε [16]. Preliminary data also indicate that Go6976, an ATP binding site inhibitor that is more selective for inhibiting PKCα, than PKCδ or PKCε [9], does not block TPA-induced aggregation of MCF10A acini (data not shown). This suggests that PKCα may not be required for this action of TPA. Furthermore, both PKCδ or PKCε can mediate changes in cell morphology and function that are associated with modulation of the actin cytoskeleton, such as migration, motility, and adhesion [28], [31]. The precise mechanisms and pathways by which the different PKC isoenzymes regulate actin dynamics in the 3D MCF10A system remains to be explored.

TPA has a biphasic effect on mitosis over the course of acinus formation. TPA blocks mitosis in the early, highly proliferative phase. Likewise, we found that TPA blocks proliferation of MCF10A cells growing in monolayer culture (data not shown). Interestingly, TPA did not block mitosis at later phases of acinus formation. The biochemical pathways that regulate the cell cycle may shift during the process of acinus formation, resulting in a change in expression of or access to molecular targets of PKC. The expression of PKC isoenzymes may also change during the course of acinus formation. Both PKCα and PKCδ can block proliferation, although PKCδ can also stimulate proliferation and death, depending on the system [5]. One limitation of phospho-histone H3 staining is that it does not directly measure cell number, such that there can be an increase in phospho-histone H3 staining, even if there is a block in cell division. Although it is possible that the increase in phospho-Histone H3 staining observed after day 6 in TPA-treated acini is accompanied by a block in cell division, we believe this is unlikely because we have not observed dramatic increases in the size of the cells in TPA-treated acini at later time points. Further research is required to determine which PKC isoenzyme is required for TPA to block mitosis, and which PKC-dependent pathways are involved in regulating the cell cycle at different phases of acinus development. Altogether, these results suggest that the effects of TPA, and activation of PKC, on the cell cycle depend not only on the cell type, but also on the biochemical and architectural state of the cell within the context of tissue development.

In contrast to mitosis, TPA did not have dramatic effects on cell death or polarization in the 3D MCF10A system. TPA did not affect the temporal regulation of cell death, although the level of cell death in TPA-treated acini was moderately higher than controls in the late phase of acini development. Debnath et al. observed that the expression of oncogenes that primarily increase cell proliferation in MCF10A cells results in a compensatory increase in apoptosis, such that the acini still developed a hollow lumen [19]. Therefore, the increased cell death observed in TPA treated acini may be a compensatory response to the burst in mitosis that follows the initial TPA-stimulated block. Some cells did remain in the lumen of TPA-treated acini, however, indicating that the increase in cell death did not totally compensate for the altered timing of mitosis. Altogether, these results indicate that abnormal activation of PKC does not directly affect cell death in the 3D MCF10A system. Finally, the cells in TPA-treated acini appeared to be polarized. Interestingly, atypical isoenzymes of PKC, which are not activated by phorbol esters, are important components of a protein complex that regulates polarity in epithelial tissues [32]. This highlights how specific isoenzymes of PKC can serve very different cellular functions.

Altogether our studies in the 3D MCF10A system, as a model of epithelial tissue, indicate that the predominant affect of TPA on the morphology of acini is due to the production of actin-based protrusions and acini aggregation, and not due to changes in the regulation of proliferation, death, polarization, and growth arrest. Our results suggest that among the many effects stimulated by the powerful tumor promoter TPA, the PKC-dependent formation of actin-containing membrane protrusions deserves attention as a potentially important trigger for events that lead to local invasion as cells progress along the pathway toward carcinogenesis.

## Supporting Information

Video S1 **Real time imaging of control cultures.** Cultures of MCF10A acini were fed on day 4. Real time imaging was started 1.5 hours later, and then continued for approximately 24 more hours. Images were captured every 10 minutes.
(AVI)

Video S2 **TPA stimulates the formation of protrusions and the aggregation of acini.** Cultures of MCF10A acini were fed on day 4 in the presence of 10 nM TPA. Real time imaging was started 1.5 hours later, and then continued for approximately 24 more hours. Images were captured every 10 minutes.
(AVI)
